# Effects of Erythropoietin-Promoted Fracture Healing on Bone Turnover Markers in Cats

**DOI:** 10.3390/jfb15040106

**Published:** 2024-04-17

**Authors:** Radina Vasileva, Tsvetan Chaprazov, Aneliya Milanova

**Affiliations:** 1 Department of Veterinary Surgery, Faculty of Veterinary Medicine, Trakia University, 6000 Stara Zagora, Bulgaria; tsvetan.chaprazov@trakia-uni.bg; 2 Department of Pharmacology, Animal Physiology, Biochemistry and Chemistry, Faculty of Veterinary Medicine, Trakia University, 6000 Stara Zagora, Bulgaria; aneliya.milanova@trakia-uni.bg

**Keywords:** erythropoietin, bone markers, comminuted fracture, femur, cats

## Abstract

In orthopaedics, erythropoietin (EPO) is applied in the preoperative management of anaemic patients, but also as a stimulating factor to assist bone regeneration due to its angiogenic and osteoinductive potential. Since orthopaedists mainly rely on their clinical experience to assess bone healing, additional and more objective methods such as studying the dynamics of bone markers are needed. Therefore, the aim of this study was to investigate the plasma activity of bone-specific alkaline phosphatase (BALP), the N-terminal propeptide of type I collagen (PINP), the C-terminal telopeptide of type I collagen (CTX), and deoxypyridinoline (DPD) during the first 2 months of healing of comminuted fractures in cats, either non-stimulated or locally stimulated with recombinant human erythropoietin (rhEPO). The study included twelve cats of mixed breeds, aged 7.2 ± 4 months, weighing 2.11 ± 1.1 kg, with comminuted diaphyseal fractures of the femur. Surgical treatment with plate osteosynthesis was performed in all animals. The cats were randomly divided into two groups—a control (*n* = 6) and an EPO group (*n* = 6). The locally applied EPO leads to the increased activity of bone formation markers (BALP and PINP) during the second week after the osteosynthesis, preceding the peaks in the control group by two weeks. The studied bone resorption markers (DPD, CTX) varied insignificantly during the studied period. In conclusion, erythropoietin could serve as a promoter of bone healing in comminuted fractures in cats.

## 1. Introduction

Erythropoietin (EPO), primarily known for its role in stimulating red blood cell production, has been found to have additional pleiotropic effects, including the promotion of tissue repair and regeneration. In bone fracture models, erythropoietin application enhances bone formation and accelerates bone healing [1]. Furthermore, EPO increases callus volume and callus mineralization, thus improving the biomechanical properties of the bone [2,3,4,5]. The exact mechanism of action is still not well established, but it is thought to be mediated by EPO receptors present on mesenchymal stem cells (MSCs). Additionally, an increase in the expression of the transcription factor Runx2 is observed, which is necessary for osteoblast growth, mineral accumulation, and the increased activity of alkaline phosphatase, osteocalcin, and bone sialoprotein [6].

In orthopaedics, EPO is applied both for the preoperative management of anaemic patients and as a drug to assist bone regeneration [7,8]. Experimental studies in mice, rats, and rabbits demonstrate its angiogenic and osteoinductive potential, manifested with the formation of new blood vessels, the differentiation of mesenchymal stem cells into osteoblasts, the regulation of the interaction between osteoblasts and osteoclasts, and enhanced bone formation [9,10]. All of the above makes EPO a key factor in strategies for bone tissue engineering, which requires the combined application of a cell source, a biomaterial or scaffold, and a biomolecule supporting the tissue [11]. Biomaterials play a crucial role in this process by providing structural support, mimicking the extracellular matrix, and promoting cellular attachment, proliferation, and differentiation. Therapeutic agents such as growth factors, peptides, cytokines or lipids are used for their ability to provide signals to cells and are also critical for tissue repair and regeneration [12]. Intensive research is underway regarding combining numerous biomaterials with erythropoietin, augmenting the osteogenesis and osteoinduction of the bone substitute. In most cases, erythropoietin is applied locally together with natural biomaterials, such as collagen or gelatin sponges being soaked with rhEpo for this purpose [13]. Cases involving its incorporation into hyaluronic acid or chitosan hydrogels to stimulate periodontal regeneration have also been described [14,15].

Lately, the focus of research is on the combined application of erythropoietin with bone grafting or biomaterials such as hydroxyapatite, tricalcium phosphate, or biphasic calcium phosphate [16,17,18,19]. Novel silk fibroin/collagen/hydroxyapatite scaffolds obtained via 3D printing technology and loaded with rhEpo have been used in the reconstruction of alveolar bone defects in rabbits [20]. The main advantage of using inorganic biomaterials, making them the most proper candidate for hard tissue engineering, is that they possess better mechanical strength than natural biomaterials and can maintain longer stability to support the early stage of the bone healing process [21]. The obtained results are promising, suggesting that the effect of their mutual application is much better than that when they are used individually. In these combinations, EPO contributes to a better result by reducing inflammation and apoptosis, promoting vascularization, facilitating endochondral ossification, and stimulating osteoblastogenesis [22]. These findings have raised the interest of clinicians seeking new approaches to stimulate bone formation, especially in cases of extensive bone defects resulting from trauma, infections, and tumours [23].

While the potential benefits of EPO in different experimental designs seem promising, clinical trials evaluating its therapeutic effect in bone tissue injuries and fracture repair are still limited. V et al. (2013) are among the first who reported accelerated bone healing following the application of a single local dose of 4000 IU recombinant human erythropoietin (rhEpo) in patients with tibiofibular fractures [24]. More recent studies suggest that EPO can be used in the non-surgical treatment of periodontitis in humans [25]. At present, there are no data regarding the application of rhEPO in clinical veterinary patients.

For the assessment of fracture healing, orthopaedists mainly rely on subjective methods such as clinical and radiological examination [26,27]. However, radiographic images can often be overestimated, necessitating additional evaluation methods. Bone turnover markers are released during the bone remodelling process. Their assay in blood allows for the tracking of bone formation and resorption during fracture healing [28], the rate of development of a bone callus, and their assay is even used as a prognostic indicator for potential complications [29,30]. Bone markers are classified depending on whether they reflect bone formation: bone-specific alkaline phosphatase (BALP), osteocalcin, the N-terminal propeptide of type I collagen (PINP), the C-terminal propeptide of type I collagen (CINP), or bone resorption processes: tartrate-resistant acid phosphatase (TRAP), the N-terminal telopeptide of type I collagen (NTX), the C-terminal telopeptide of type I collagen (CTX), and deoxypyridinoline (DPD). 

In human medicine, bone markers are primarily used for diagnosing and monitoring osteoporosis and Paget’s disease, which are the most common metabolic bone disorders [31,32]. They can also serve as prognostic indicators for certain bone-affecting tumour formations, including those of the breast, prostate, and multiple myeloma [33,34,35]. In recent years, changes in bone marker levels have been studied in rheumatoid arthritis and ankylosing spondylitis, where the bone formation is suppressed, and bone resorption takes precedence [36,37]. Just as important, few authors have examined bone turnover markers as predictors of a normal fracture healing process or a delayed union [38,39].

In veterinary medicine, bone markers are used as a rapid and sensitive method to track the response to treatment or surgical intervention in horses [40,41], sheep [42,43], dogs [44], and cats [45,46,47]. Reference values for bone markers in dogs, in relation to their age [44] and breed [48], are reported in the literature. Bone metabolism markers have been studied in metabolic bone diseases [49], osteomyelitis [50], bone tumours [51], and various experimental fracture models and bone defects [52]. 

Studies analysing bone markers in relation to fracture healing in cats are very few. Therefore, the aim of this study was to investigate the activity of bone-specific alkaline phosphatase (BALP), the N-terminal propeptide of type I collagen (PINP), the C-terminal telopeptide of type I collagen (CTX), and deoxypyridinoline (DPD) in blood plasma during the non-stimulated or the locally stimulated-with-recombinant-human-erythropoietin healing of comminuted fractures in cats.

## 2. Materials and Methods

### 2.1. Study Cohort

The study included twelve cats of mixed breeds, aged 7.2 ± 4 months and weighing 2.11 ± 1.1 kg. All were clinical patients at the Small Animal Clinic of the University Veterinary Hospital, Faculty of Veterinary Medicine, Trakia University, diagnosed with comminuted diaphyseal fractures of the femur. The diagnosis was made after the clinical examination and radiography of the affected limb. The cats were randomly divided into two groups—a control (*n* = 6) and an EPO group (*n* = 6). For all the patients, surgical treatment was performed. The protocol was approved by the Ethics Committee of the Faculty of Veterinary Medicine, Trakia University (Approval code 02 and Approval Date 2 June 2021). The owners of the cats included in the study have signed informed consent forms about the objectives of the study. 

Inclusion criteria comprised cats with a recent comminuted fracture of the femoral diaphysis. Exclusion criteria included: animals with systemic diseases; animals with multiple fractures and animals which, due to the type of fracture, needed a combination of different techniques of bone fixation at the same time. 

### 2.2. Anesthesia and Surgical Procedure 

The cats were premedicated with atropine sulfate (0.1% Atropin, Sopharma, Sofia, Bugaria) at a dose of 0.02 mg/kg, applied subcutaneously. The induction of anaesthesia was done via the intravenous application of the combination of zolazepam/tiletamine (Zoletil 50, Virbac, Carros, France) at a dose of 15 mg/kg, and the maintenance—through inhalational anaesthesia with 2.5% isoflurane with oxygen (AErrane, Baxter, Deerfield, IL, USA). Fluid management included a Ringer lactate infusion at a rate of 10 mL/kg/h. 

For surgery, cats were positioned in lateral recumbency with the affected limb exposed. After a lateral approach to the femur, the bone fragments were reduced and fixed with a metal plate and screws. In the EPO group, erythropoietin (Binocrit 2000 IU, Sandoz GmbH, Basel, Switzerland) was locally applied at the fracture site at a dose of 1000 IU, equivalent to 0.5 mL of solution. In the control group, the same amount of a saline solution was applied. The determination of the appropriate dose of rhEpo for cats was based on the fact that in this species, erythropoietin alpha is administered at a therapeutic dose of 100 IU/kg three times a week. Typically, the medication is administered subcutaneously, whereas in this experiment, it was applied locally at the site of the fracture line to avoid the potential side effects associated with systemic EPO treatment.

During the post-operative period, pain was controlled with meloxicam (Meloxidolor, Produlab Pharma, Raamsdonksveer, The Netherlands) at a dose of 0.3 mg/kg, applied subcutaneously. Infection was prevented via the subcutaneous administration of 25 mg/kg amoxicillin/clavulanic acid (Synulox RTU, Pfizer, New York, NY, USA) for 6 days.

### 2.3. Radiographic Analysis

The radiological examinations of the femoral region were performed using an X-ray system (PHILIPS SUPER 50 CP-D, Hamburg, Germany) before the surgery, immediately after the surgical repair, and at postoperative weeks 2, 4, and 8. The radiography parameters were 50 kV and 10 mAs. The data were processed by iQ-VIEW/PRO version 2.7.0 software. The researcher who scored all radiographs with 1 to 6 points (0: the presence of a fracture line with no bone formation; 1: a visible fracture line, no callus formation; 2: a visible fracture line with initial callus formation; 3: a visible fracture line with bridging callus formation; 4: an exuberant osseous callus in evolution and a discrete radiolucent line at the gap between the fracture fragments; 5: an exuberant osseous callus and an absence of a radiolucent line; 6: a bone remodelling that was blind to the given treatment (EPO or control).

### 2.4. Analysis of Bone Turnover Markers in Blood Plasma 

To determine the concentrations of bone markers, blood samples were obtained in EDTA tubes from the cephalic vein of the cats on the day of the surgical intervention and at the 2nd, 4th, and 8th weeks postoperatively. To eliminate circadian changes, blood collection was performed between 7 and 8 A.M. After obtaining the blood samples within the mentioned intervals, the samples were centrifuged for 10 min at 3000 rpm to obtain an EDTA plasma, which was stored at −80 °C until the time of analysis. 

The following bone markers (in ng/mL) were assayed using commercially available enzyme-linked immunosorbent assay kits: Cat Bone-specific Alkaline Phosphatase (BALP, BT LAB, Birmingham, UK); Cat Total Procollagen Type I intact N-terminal Propeptide (TP1NP, BT LAB, Birmingham, UK); Cat Cross-Linked C-terminal telopeptides of Type I Collagen (CTX-I, Abebio, Wuhan, China), and Cat Deoxypyridinoline (DPD, Abebio, Wuhan, China). 

### 2.5. Statistical Analysis

The statistical analysis of the changes in bone markers with time in both groups was performed using a repeated-measures ANOVA. Between-group comparisons at each post-fracture time interval were completed using an independent *t*-test. The associations between the radiological scoring and bone markers measurements were analysed using Spearman’s rank order correlation coefficient (rho). All analyses were performed on MedCalc Software (version 15.8, Ostend, Belgium).

## 3. Results

The radiological scores are shown in Table 1. During the second week after surgery, the beginning of bone callus formation was observed, which was significantly more pronounced in the EPO group. By the fourth week, bone bridging between the fragments was established, with a statistically significantly higher score in the EPO group. Bone remodelling was observed at the 8th week in both groups.

**Figure 1:**
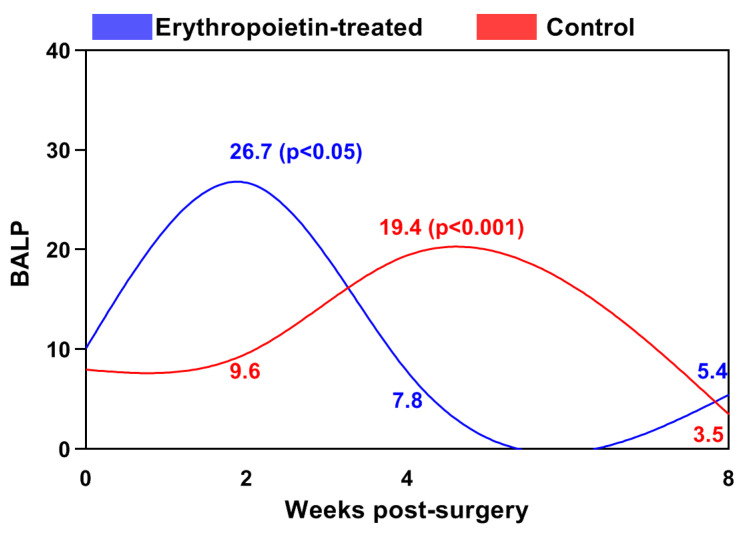
Changes in the blood levels of bone-specific alkaline phosphatase (BALP, ng/mL) in the cats from both groups.

The activity of bone-specific alkaline phosphatase (BALP) in the control group gradually increased during the first two weeks after the surgical intervention, reaching its peak in the fourth week, after which it significantly decreased until the end of the study (Figure 1). The difference in the values at the fourth week was statistically significant (*p* < 0.001) compared to those of the initial period. 

In the EPO group, this peak occurred as early as the second week (26.72 ± 13.77 ng/mL), when BALP values were two and a half times higher than those of the baseline level. From the second to the eighth week, its activity significantly decreased. The between-group differences were statistically significant by the 2nd week (*p* < 0.05) and the 4th post-operative weeks (*p* < 0.001). The values were higher at the 2nd week and lower at the next interval in the EPO group.

**Figure 2:**
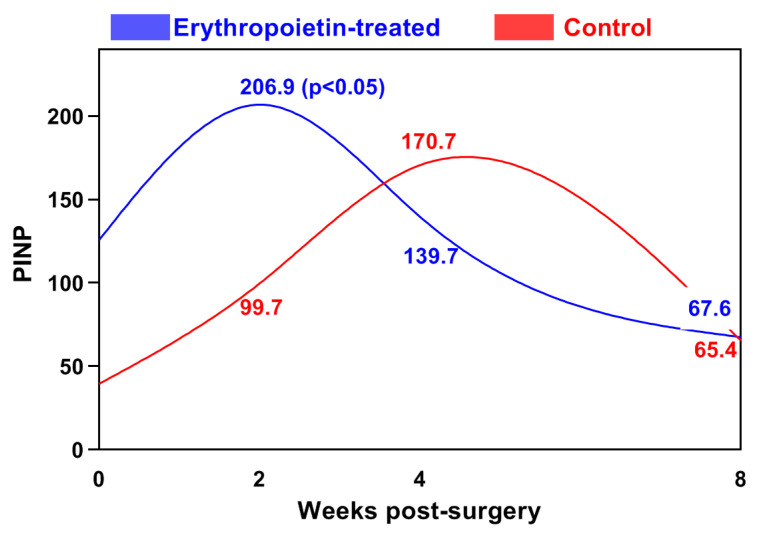
Changes in the blood levels of the N-terminal propeptide of type I collagen (PINP, ng/mL) in the cats from both groups.

The N-terminal propeptide of type I collagen (PINP) showed a tendency for gradual increase, reaching its peak at the fourth week in the control group (170.60 ± 55.72 ng/mL). Its values were significantly higher at the 2nd (*p* < 0.05) and 4th (*p* < 0.001) weeks compared to those of the baseline level, after which they decreased until the end of the study (Figure 2).

In the EPO group, the highest PINP concentration (206.87 ± 106.87 ng/mL) was measured during the second week following the surgical intervention. The difference between the two groups at this time interval was statistically significant (*p* < 0.05). 

**Figure 3:**
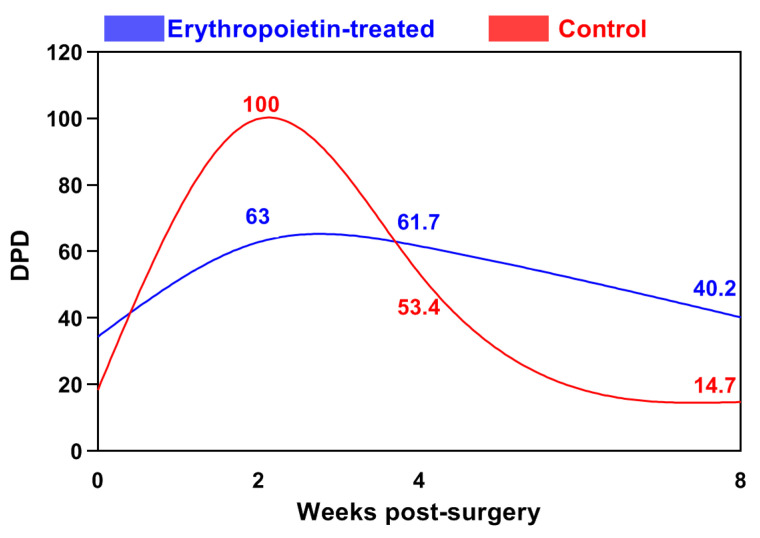
Changes in the blood levels of deoxypyridinoline (DPD, ng/mL) in the cats from both groups.

In the control group, the blood deoxypyridinoline (DPD) increased significantly compared to that of the baseline level (18.38 ± 3.13 ng/mL), reaching a peak in the second week after osteosynthesis (99.93 ± 36.42 ng/mL) (Figure 3). The observed difference was statistically significant (*p* < 0.001). Following this peak, the DPD values declined, reaching 53.43 ± 5.44 ng/mL in the fourth week (*p* < 0.05).

In the EPO group, DPD levels increased gradually until the second week, remained stable in the fourth week, and did not demonstrate any statistically significant changes until the end of the second month.

**Figure 4:**
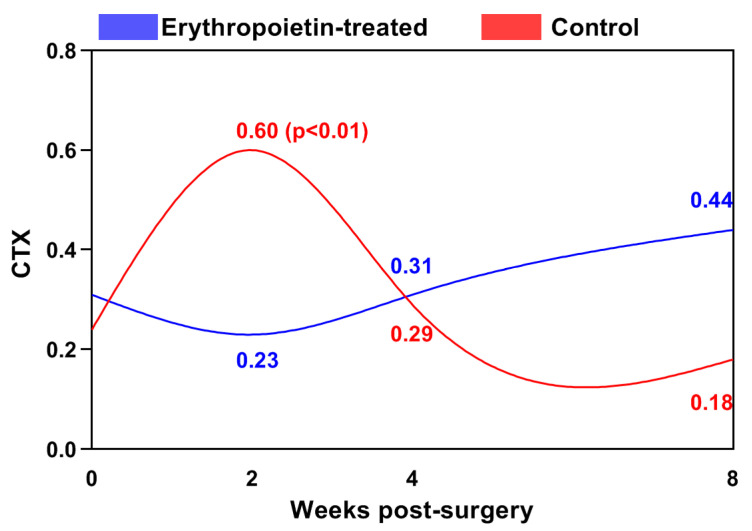
Changes in the blood levels of the C-terminal telopeptide of type I collagen (CTX, ng/mL) in the cats from both groups.

The levels of the C-terminal telopeptide of type I collagen (CTX) in the control group reached a peak of 0.60 ± 0.19 ng/mL (*p* < 0.001 vs. baseline) during the second week, and decreased gradually until the end of the study (Figure 4). 

In the EPO group, the blood CTX concentrations were the lowest in the second week (0.23 ± 0.17 ng/mL), after which they insignificantly increased until the eighth week. In the second week, statistically significantly lower levels of CTX were observed in the EPO group than in the control group (*p* < 0.01).

The radiological score by the 2nd week demonstrated a statistically significant inverse association with BALP concentrations by the 4th week (−0.751; *p* < 0.01). The radiological score by the 4th week correlated positively with the BALP at 2 weeks (r = 0.662, *p* < 0.05) and the PINP at 2 weeks (r = 0.74, *p* < 0.01) (Table 2). Regarding the bone resorption markers (DPD and CTX), no significant correlations with radiological scores were found.

## 4. Discussion

The healing of comminuted fractures takes more time and is much more likely to result in complications. The key to their treatment is the proper alignment of the fragments, their stable fixation, and their having an adequate blood supply [53]. Data from recent studies demonstrate that in addition to its haematopoietic effects, erythropoietin also possesses osteogenic and angiogenic potential. EPO appears to enhance the recruitment and differentiation of mesenchymal stem cells into osteoblasts [54]. In addition, EPO has demonstrated anti-inflammatory properties. Inflammatory responses play a crucial role in the early stages of bone repair, and the modulation of these responses by EPO could promote a more favourable environment for healing [55]. Just as importantly, EPO has angiogenic effects, promoting the formation of new blood vessels. An adequate blood supply is essential for bone healing, as it delivers oxygen, nutrients, and progenitor cells to the site of the injury [56]. Taking everything into consideration, EPO treatment may enhance bone healing. After the report of Bakhshi et al. (2013) regarding accelerated bone healing in patients with tibiofibular fractures following a local injection of erythropoietin, our interest in the possible positive effect of EPO on bone healing promotion in cats has increased significantly [24].

Bone healing is characterised by increased osteoblastic activity, as detected by the examination of bone markers in blood serum or plasma. Osteoblasts, responsible for both the formation of new tissue (bone matrix) and its mineralization, secrete large amounts of alkaline phosphatase (ALP), which participates in this process [57]. In humans and animals, alkaline phosphatase isoenzymes originate from the liver, intestines, placenta, kidneys, and bones [58]. Our attention was focused on bone alkaline phosphatase (BALP) as it is considered to be more specific [59]. An increase in BALP activity was observed in the second week after osteosynthesis compared to that at the baseline level. Similar early increases in ALP levels have also been observed in dogs with fractures [60]. In other human studies, serum ALP activity began to increase 7–9 days after the fracture, and the increase was significant at around 4 or 6 weeks [61]. These findings are comparable with the data observed in our control group, where the peak of bone-specific alkaline phosphatase occurred in the fourth week after osteosynthesis. However, in the EPO group, this peak was observed as early as the second week. This earlier increase in BALP activity, in our opinion, was due to the erythropoietin-stimulated early osteoblastogenesis. In vitro studies using human and mouse bone mesenchymal stem cells (BMSCs) treated with EPO reported increased osteoblast differentiation with the activation of EphrinB2/EphrinB4 [62], mTOR [63], and JAK2/PI3K pathways [64]. An alternative mechanism, in which EPO stimulates hematopoietic stem cells to produce bone morphogenetic proteins, which in turn stimulate osteoblasts, is also possible [10]. According to Balaian et al. (2018), EPO’s bone remodelling effect could be age-dependent, resulting in greater mineralization only in young, healthy patient’s cells [65]. Additionally, in contrast to young mice, EPO may not be used to improve bone healing in the elderly [66].

Another marker of the bone formation made in the osteoblast and secreted into the new bone matrix is the N-terminal propeptide of type I collagen (PINP). According to Coulibaly et al. (2010), quantitative changes in the serum PINP are significantly more specific and indicative than changes in alkaline phosphatase or osteocalcin under conditions of increased osteogenic activity [67]. This was the reason we chose it as the second marker of bone formation. It has been found that PINP increased during the early period after the fracture, reaching a peak in the second week, after which it returned to its baseline level within 4 to 6 months [68,69]. Coulibaly et al. (2010) observed a PINP peak corresponding to the period of callus formation, during which PINP is primarily released; these results are confirmed by our study as well [67]. 

The results of the correlation analysis indicate that there was a significant positive correlation between the concentrations of BALP and PINP in the second week and the radiographic scores by the fourth weeks. The activity of the bone markers responsible for bone formation precedes the radiological appearance of the bone callus by two weeks. This is based on the fact that molecular level changes need to occur first, and they only later become visible on radiographic images [70].

The specificity of deoxypyridinoline (DPD) in fracture healing is still not well studied [71]. It is produced from collagen degradation, thus reflecting bone resorption processes [72]. Our results showed that in the EPO group, its levels decreased in the second week after the surgical intervention, after which they gradually increased and remained relatively constant until the end of the study, corresponding to bone remodelling. In the control group, there was a significant increase in the second week compared to that of the baseline, likely due to an enhanced resorption at the bone ends of the fracture site.

Variations in the levels of the C-terminal telopeptide of type I collagen (CTX) resemble the processes of bone resorption documented in experimental studies of fractures in dogs using two different osteosynthesis techniques [73]. Concentrations of CTX in the mentioned study increased significantly compared to those at baseline until the first month after surgery. According to the authors, CTX was a reliable indicator in monitoring normal bone healing, as changes in its concentration corresponded to the resorption at the bone ends immediately after the trauma (osteotomy or fracture). Theyse et al. (2006) reported that even the placement of a fixation device without osteotomy triggered a bone reaction and led to an early (up to the second week) increase in concentrations of the C-terminal telopeptide of type I collagen, as observed in our control group [74]. In the EPO group, an opposite trend was noticed, with CTX values gradually decreasing until the second week, reaching their peak in the eighth week after surgery. The increase at a later stage corresponds to the moment when the newly formed bone callus undergoes remodelling. This event occurs at a different time when different osteosynthesis techniques are used [73].

The influence of EPO on osteoclasts remains contradictory. Few studies have reported that EPO directly stimulates osteoclast precursors and induces bone loss [75]. Increased bone resorption and a decreased bone healing rate are observed in murine transgenic models of EPO overexpression [76]. It appears that this effect is dose-dependent and associated with a higher dose of EPO [77]. Other authors report that EPO increases the number of osteoclasts, but does not affect their activity [6,62]. This statement is indirectly supported by our results, which did not establish a statistically significant difference in the products of osteoclastic activity, namely DPD and CTX, between the EPO and the control group.

Contrary to our results, Nelson et al. (2005) did not observe changes in the bone turnover markers in recreational athletes following the administration of rhEpo [78]. The likely reason is due to the fact that during fracture healing, bone marker activity is several times higher compared to those of the physiological remodelling cycle [79]. Secondly, as mentioned above, EPO’s effect is age-dependent, appearing to have an anabolic effect on bone healing in our feline patients, who are still growing.

This study reflects the changes in bone metabolism during the early stage of the erythropoietin-promoted healing of fragmented fractures in cats and some potential limitations should be taken into account when interpreting the obtained results. As a pilot investigation, the results can serve to generate hypotheses. One of this study’s main limitations is the small number of patients included. It is necessary to expand the studies among the cat population to analyse differences in the levels of bone markers in relation to a cat’s sex, breed, and age.

## 5. Conclusions

In cats with fragmented fractures of the femur, locally applied erythropoietin led to a greater plasma concentration of bone formation markers (BALP and PINP) in the second week after osteosynthesis, which preceded the peak in the untreated control group by two weeks. This indicated an increased erythropoietin-promoted osteoblast activity during the acute fracture healing stage.

## Figures and Tables

**Table 1:** Radiological healing scores in cats from the control (*n* = 6) and EPO-treated (*n* = 6) groups. Data are presented as medians (min–max).

Post-Operative Week	Control Group	EPO Group
2 w	2 (2–3)	3 (2–30) *
4 w	3.5 (3–4)	4.5 (4–5) *
8 w	6 (5–6)	6 (6–6)

* *p* < 0.05 between groups.

**Table 2:** Spearman correlation coefficients between the radiological scores of the 2nd week (2 w) and those of the 4th week (4 w), and the bone formation markers at the same time intervals.

		BALP (2 w)	BALP (4 w)	PINP (2 w)	PINP (4 w)	RO (2 w)	RO (4 w)
BALP (2 w)	Correlation coefficient						
	Significance level *p*						
	*n*						
BALP (4 w)	Correlation coefficient	−0.336					
	Significance level *p*	0.3118					
	*n*	12					
PINP (2 w)	Correlation coefficient	0.918	−0.355				
	Significance level *p*	0.0001	0.2847				
	*n*	12	12				
PINP (4 w)	Correlation coefficient	0.282	0.591	0.382			
	Significance level *p*	0.4011	0.0556	0.2466			
	*n*	12	12	12			
RO (2 w)	Correlation coefficient	0.231	−0.751	0.115	−0.577		
	Significance level *p*	0.4945	0.0078	0.7353	0.0629		
	*n*	12	12	12	12		
RO (4 w)	Correlation coefficient	0.662	−0.584	0.740	0.039	0.236	
	Significance level *p*	0.0266	0.0593	0.0093	0.9095	0.4608	
	*n*	12	12	12	12	12	

## Data Availability

Data can be obtained from the corresponding author upon reasonable request.
